# Multimodal MRI of choroid plexus volume, DTI-ALPS, and white matter microstructure in type 2 diabetes mellitus

**DOI:** 10.3389/fmed.2026.1968119

**Published:** 2026-10-07

**Authors:** Minghui Mao, Linghan Chen, Juan Xia, Taipeng Zeng, Hao Huang, Jianfeng Chu, Liyuan Fu

**Affiliations:** 1 Fuzong Teaching Hospital of Fujian University of Traditional Chinese Medicine (900th Hospital), Fujian, Fuzhou, China; 2 Department of Radiology, 900th Hospital of PLA Joint Logistic Support Force, Fujian, Fuzhou, China; 3 College of Integrative Medicine, Fujian University of Traditional Chinese Medicine, Fujian, Fuzhou, China

**Keywords:** choroid plexus, DTI-ALPS, perivascular space, PSMD, type 2 diabetes mellitus

## Abstract

**Objective:**

To investigate differences in choroid plexus volume (CPV), diffusion tensor image analysis along the perivascular space (DTI-ALPS) indices, peak width of skeletonized mean diffusivity (PSMD), and Mini-Mental State Examination (MMSE) scores between patients with type 2 diabetes mellitus (T2DM) and healthy controls, and to explore pairwise associations among diabetes duration, PSMD, normalized total CPV, total DTI-ALPS, and MMSE within the T2DM group.

**Methods:**

This retrospective cross-sectional study included 31 patients with T2DM and 31 healthy controls (HCs). All participants underwent 3.0-T MRI and MMSE assessment. General linear models were used to compare normalized CPV, DTI-ALPS indices, PSMD, and MMSE scores between groups, adjusting for age, sex, body mass index, and years of education. Within the T2DM group, exploratory partial correlations were performed among diabetes duration, PSMD, normalized total CPV, total DTI-ALPS, and MMSE, controlling for the same covariates. The resulting 10 pairwise associations were corrected together using the Benjamini-Hochberg false discovery rate (FDR) procedure.

**Results:**

Compared with HCs, patients with T2DM showed greater normalized total and bilateral CPV, lower total DTI-ALPS, higher PSMD, and lower MMSE scores (all *P_FDR_* < 0.05). Among the 10 exploratory associations, only the positive association between diabetes duration and PSMD remained significant after FDR correction (*partial r* = 0.566, *P* = 0.002, *P_FDR_* = 0.021). The inverse association between normalized total CPV and total DTI-ALPS was nominally significant (*partial r* = −0.412, *P* = 0.033) but did not survive FDR correction (*P_FDR_* = 0.163).

**Conclusion:**

Patients with T2DM showed differences in CPV, DTI-ALPS, PSMD, and MMSE scores. Longer diabetes duration was associated with higher PSMD after FDR correction, whereas the CPV–DTI-ALPS association did not survive multiple-comparison correction. These findings highlight the value of multimodal MRI for characterizing brain involvement in T2DM.

## 1 Introduction

Type 2 diabetes mellitus (T2DM) is a systemic metabolic disorder characterized by insulin resistance, pancreatic *β*-cell dysfunction, and chronic hyperglycemia (1). Beyond its well-established peripheral complications, T2DM is increasingly recognized to involve the central nervous system, with alterations in brain structure, white matter microstructure, and cognitive function consistently reported across studies (2, 3). Metabolic dysregulation, cerebral microvascular dysfunction, chronic inflammation, and blood–brain barrier disruption have all been implicated in these diabetes-related brain changes (2–4). However, these imaging features have largely been investigated in isolation, and their interrelationships remain insufficiently characterized.

**Table 1:** Comparison of demographic, clinical, and cognitive characteristics between the two groups.

Characteristic	HC (*n* = 31)	T2DM (*n* = 31)	*P*
Sex (male)	14 (45.2%)	14 (45.2%)	1.000
Age (years)	57.23 ± 5.91	61.03 ± 8.88	0.052
BMI (kg/m^2^)	24.41 ± 3.52	23.19 ± 2.46	0.118
Years of education (years)	9.00 (7.00, 11.00)	7.00 (6.00, 11.00)	0.323
FBG (mmol/L)	5.20（4.85, 5.43）	8.60（7.35, 11.93）	<0.001
Diabetes duration (years)	—	10.00 (9.00, 15.00)	—
HbA1c (%)	—	8.80 (7.70, 10.40)	—
MMSE score	28.00 (26.00, 29.00)	25.00 (23.00, 27.00)	<0.001
TIV (cm^3^)	1,452.48 ± 99.75	1,444.10 ± 116.46	0.762

Categorical variables are presented as frequencies (percentages), and continuous variables as mean ± standard deviation or median (interquartile range), as appropriate. Between-group comparisons used the chi-square test, independent-samples t test, or Mann–Whitney U test, as appropriate. T2DM, type 2 diabetes mellitus; HC, healthy control; BMI, body mass index; FBG, fasting blood glucose; HbA1c, glycated hemoglobin; MMSE, Mini-mental State Examination; TIV, total intracranial volume. —, not applicable. P values are unadjusted

White matter microstructural alterations are among the most consistently reported neuroimaging findings in T2DM. Diffusion imaging studies have demonstrated widespread white matter abnormalities, with several DTI- and NODDI-derived measures showing associations with diabetes duration and metabolic control (4–6). Large-scale studies have further linked longer disease duration and higher HbA1c levels to abnormalities across multiple diffusion metrics. Peak width of skeletonized mean diffusivity (PSMD) is an automated whole-brain DTI-derived measure that quantifies the width of the mean diffusivity distribution within the white matter skeleton, thereby capturing heterogeneity in white matter diffusion (7, 8). Despite its potential sensitivity to diffuse white matter injury, PSMD has been relatively understudied in T2DM. A recent study reported elevated PSMD in patients with T2DM and mild cognitive impairment and further showed that higher PSMD was associated with longer diabetes duration and poorer cognitive performance (9).

Beyond white matter microstructure, T2DM may also involve imaging features related to brain fluid homeostasis. The glymphatic system facilitates exchange between cerebrospinal fluid (CSF) and interstitial fluid and contributes to the clearance of metabolic waste from the brain (10). Diffusion tensor image analysis along the perivascular space (DTI-ALPS), derived from directional white matter diffusivity, has been proposed as an indirect MRI marker related to glymphatic processes (11). However, DTI-ALPS does not directly quantify glymphatic flow or CSF–interstitial fluid exchange and may also be influenced by local fiber architecture, white matter microstructure, vascular lesions, and partial-volume effects. Reduced DTI-ALPS indices have been reported in patients with T2DM (12–15). The choroid plexus, meanwhile, plays a central role in CSF production, maintenance of the blood–CSF barrier, and neuroimmune regulation (16, 17), and recent studies have reported greater choroid plexus volume (CPV) in T2DM (18, 19). Although CPV and DTI-ALPS reflect distinct structural and diffusion-related imaging characteristics, both have been examined in the context of intracranial fluid homeostasis. An inverse association between CPV and DTI-ALPS has previously been reported in overt hypothyroidism (20), but whether a similar relationship is present in T2DM remains unclear.

Accordingly, the present study aimed to characterize T2DM-related brain differences across three imaging domains: choroid plexus structure, perivascular diffusion-related characteristics, and white matter microstructure. We compared normalized CPV, DTI-ALPS indices, PSMD, and MMSE scores between patients with T2DM and healthy controls. Within the T2DM group, we further explored pairwise associations among diabetes duration, PSMD, normalized total CPV, total DTI-ALPS, and MMSE. We hypothesized that patients with T2DM would exhibit greater CPV, lower DTI-ALPS indices, and higher PSMD than healthy controls; MMSE scores were additionally examined as a cognitive screening measure.

## 2 Methods

Figure 1 summarizes the study design, including the participant screening and exclusion process (Figure 1A) and the overall study workflow and analytical framework (Figure 1B).

**Figure 1:**
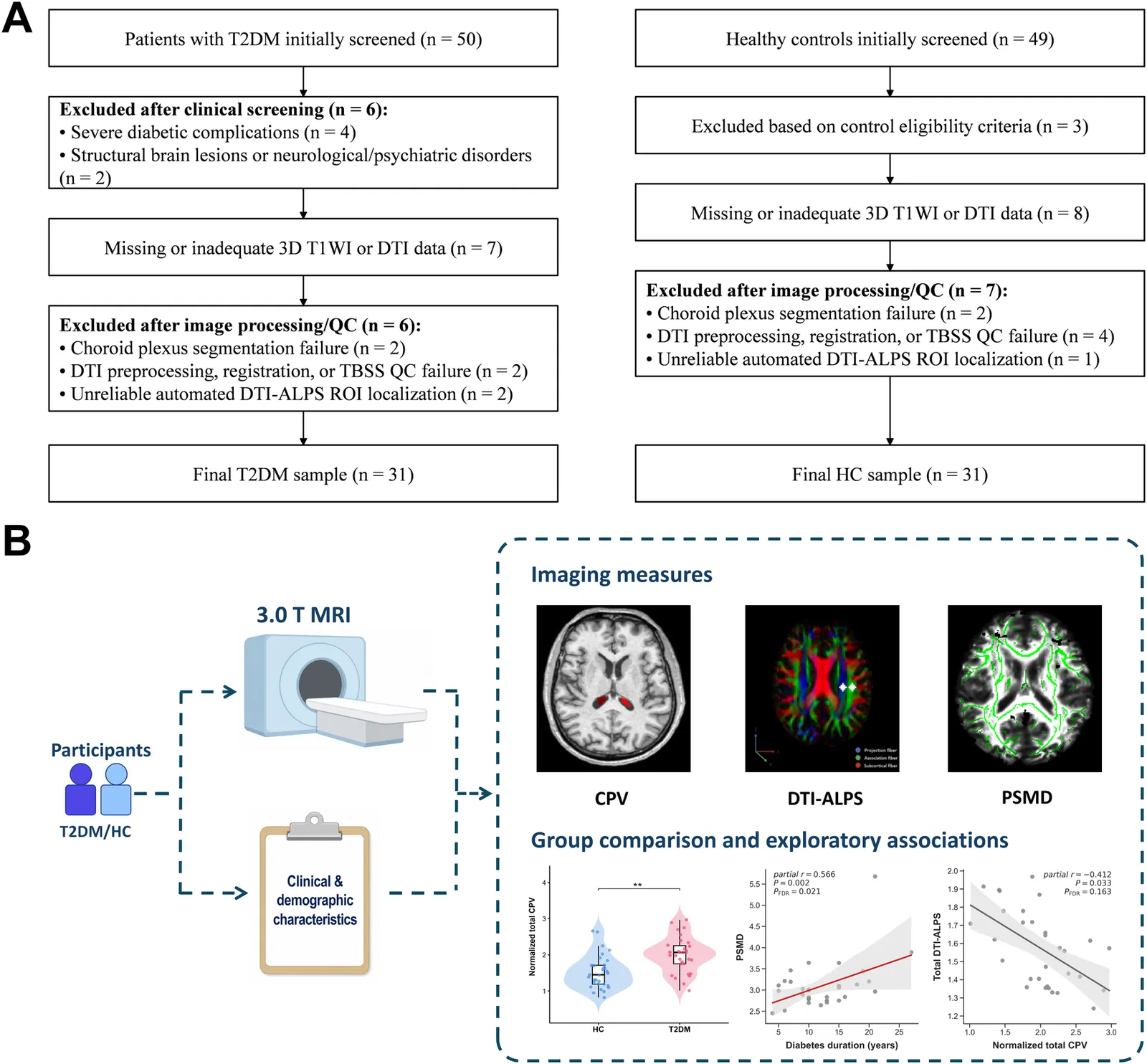
Study workflow and analytical framework. **(A)** Participant screening and exclusion process. **(B)** Overview of MRI acquisition, clinical and demographic assessment, imaging measures, and statistical analyses, including representative group comparisons and exploratory associations. CPV measures were normalized as (CPV/TIV) × 10^3^. The resulting normalized CPV values are dimensionless.

### 2.1 Study participants

This retrospective cross-sectional study included patients with T2DM who were treated in the Department of Endocrinology at the 900th Hospital of the Joint Logistics Support Force between May 2014 and January 2015, as well as healthy controls (HCs) who were concurrently recruited from the health examination center of the same hospital. T2DM was diagnosed by endocrinologists according to the World Health Organization criteria for the diagnosis and classification of diabetes mellitus (21). HCs were required to have no history of glucose intolerance or family history of diabetes and a fasting blood glucose (FBG) level <7.0 mmol/L.

The exclusion criteria were as follows: (1) missing or inadequate three-dimensional T1-weighted imaging (3D T1WI) or diffusion tensor imaging (DTI) data; (2) severe diabetic complications, such as stage III or more advanced diabetic nephropathy or recent diabetic ketoacidosis, in patients with T2DM; (3) structural brain lesions, a definite history of traumatic brain injury, or documented neurological, psychiatric, or neurocognitive disorders based on available medical records and routine clinical history; and (4) unreliable imaging results due to failures in image segmentation, DTI preprocessing or registration, TBSS quality control, or automated DTI-ALPS ROI localization. No dedicated neurological, psychiatric, or neurocognitive diagnostic assessments were performed specifically for study eligibility; therefore, unrecognized neurological or neurocognitive comorbidities could not be excluded.

All participants were right-handed and underwent 3.0-T MRI using a standardized protocol and MMSE assessment. No specific MMSE cutoff or cognitive-performance threshold was used for participant exclusion, because the MMSE was used as a cognitive screening measure rather than as an eligibility criterion. The final analytic sample comprised 31 patients with T2DM and 31 HCs. Imaging data were used to derive choroid plexus volume (CPV), peak width of skeletonized mean diffusivity (PSMD), and DTI-ALPS measures, while the MMSE was used as a global cognitive screening measure.

The study was approved by the Biomedical Ethics Committee of the 900th Hospital of the Joint Logistics Support Force (approval no. 2026-043). Because of the retrospective design and the use of fully anonymized data, the requirement for written informed consent was waived by the ethics committee.

### 2.2 Clinical and cognitive assessments

Demographic information, including age, sex, and years of education, was collected for all participants, and diabetes duration was recorded for patients with T2DM. Height and weight were measured, and body mass index (BMI, kg/m^2^) was calculated. Fasting blood glucose (FBG) was recorded for all participants, and glycated hemoglobin (HbA1c) was recorded for patients with T2DM. Cognitive screening was performed in all participants using the Mini-Mental State Examination (MMSE) (22).

### 2.3 MRI data acquisition

All MRI data were acquired on a 3.0-T superconducting MRI scanner (Siemens Magnetom Trio Tim, Erlangen, Germany) using a standard phased-array head coil. Participants’ heads were stabilized during scanning, and noise-reduction measures were used to minimize head motion and scanner noise. Conventional T2-weighted imaging (T2WI) and diffusion-weighted imaging (DWI) were acquired to exclude obvious structural brain abnormalities.

Whole-brain 3D T1-weighted images were acquired using a magnetization-prepared rapid acquisition gradient-echo (MP-RAGE) sequence with the following parameters: repetition time (TR) = 1900ms, echo time (TE) = 2.52 ms, flip angle = 9°, matrix = 256 × 256, field of view (FOV) = 250 × 250 mm^2^, slice thickness = 1.0 mm, interslice gap = 0 mm, number of excitations (NEX) = 1, and acquisition time = 258 s. A total of 176 sagittal slices were acquired to cover the whole brain. DTI was acquired using a single-shot spin-echo echo-planar imaging (SE-EPI) sequence with the following parameters: TR = 7200 ms, TE = 104 ms, flip angle = 90°, matrix = 128 × 128, FOV = 240 × 240 mm^2^, slice thickness = 3.0 mm, and interslice ga*p* = 0 mm. Diffusion-sensitizing gradients were applied along 64 directions at a b value of 1,000 s/mm^2^, together with one non-diffusion-weighted image at b = 0 s/mm^2^. The phase-encoding direction was anterior-to-posterior (A-P). No reversed phase-encoding b0 images or field map were acquired.

### 2.4 Image processing and metric extraction

#### 2.4.1 T1-weighted image processing and choroid plexus volume quantification

The choroid plexus was automatically segmented using the 3D U-Net–based model developed by Eisma et al. (23), implemented through a Docker-based version of the publicly available ChP-Seg pipeline. The model had been previously validated against manual reference segmentations in the original study. Each participant's 3D T1-weighted image was first nonlinearly registered to the ICBM-MNI 152 standard space. A region containing the choroid plexus was then cropped according to a probabilistic atlas derived from manual reference segmentations and entered into the pretrained segmentation model. The resulting segmentation mask was restored to the original image dimensions and inverse-transformed to the participant's native space for volumetric quantification. Representative native-space segmentation results are shown in Figure 2.

**Figure 2:**
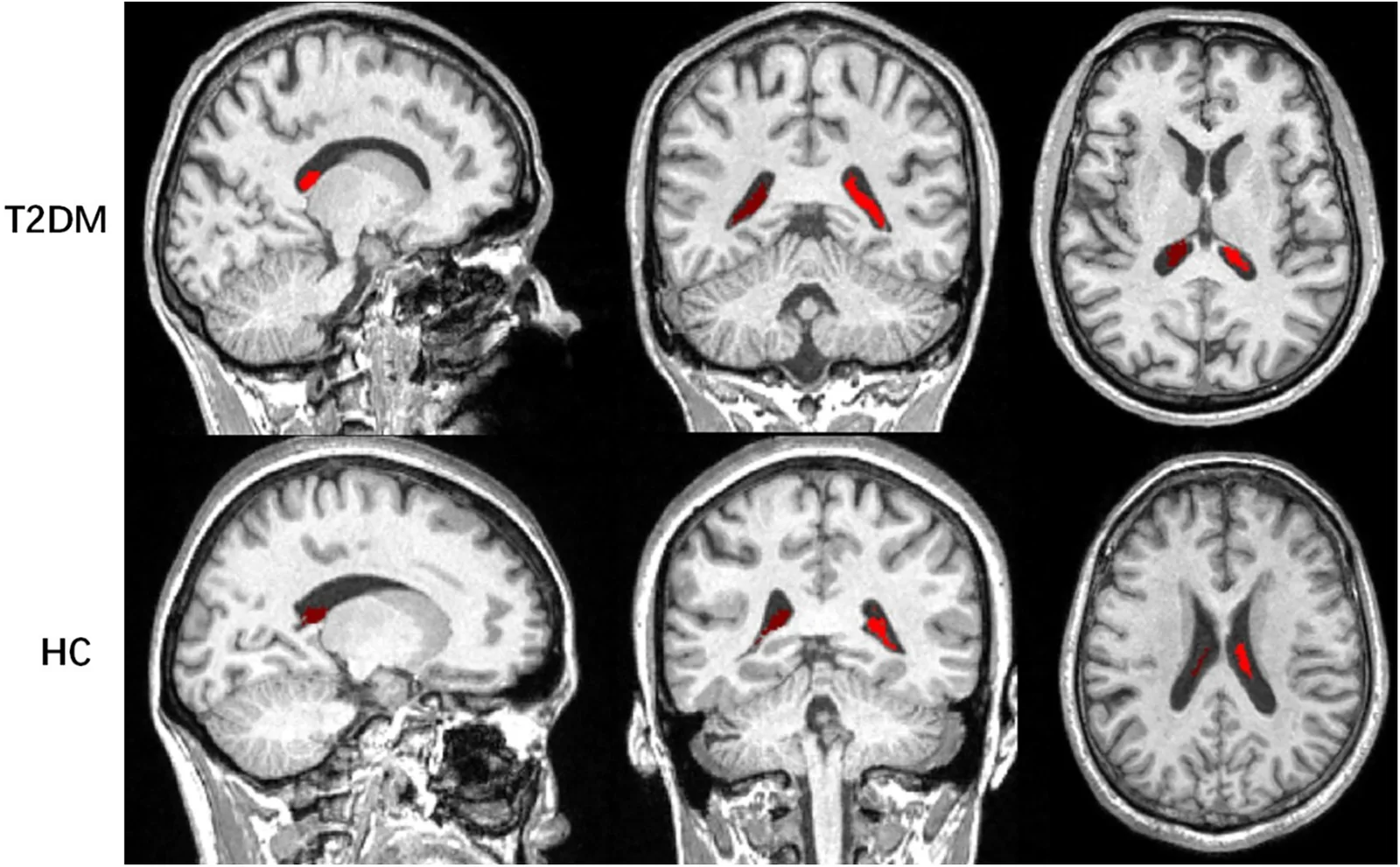
Representative choroid plexus segmentation in patients with type 2 diabetes mellitus and healthy controls. Sagittal, coronal, and axial views of native-space 3D T1-weighted images from representative participants with type 2 diabetes mellitus (T2DM) and healthy controls (HCs) are shown. Red regions indicate the automatically segmented choroid plexus within the lateral ventricles using the 3D U-Net–based ChP-Seg model.

Segmentation was restricted to the choroid plexus within the bilateral lateral ventricles; choroid plexus within the third and fourth ventricles was not included. Left and right choroid plexus volumes were calculated separately, and total choroid plexus volume (CPV) was defined as the sum of the left and right lateral ventricular choroid plexus volumes. Total intracranial volume (TIV) was obtained using the standard recon-all pipeline in FreeSurfer version 8.1. To account for individual differences in head size, CPV measures were normalized as (CPV/TIV) × 10^3^.

Two researchers with experience in neuroimaging independently performed visual quality control of the native-space segmentation masks to assess anatomical correspondence and identify tissue omissions or missegmentations. Both QC readers were blinded to group allocation. Disagreements were resolved by consensus, and cases with evident segmentation failure were excluded. No manual correction of the segmentation masks was performed. Four participants (2 with T2DM and 2 HCs) were excluded because of evident choroid plexus segmentation failure identified during visual quality control.

#### 2.4.2 DTI data preprocessing

DTI data were first converted from DICOM to NIfTI format, with corresponding bval and bvec files generated, and were subsequently preprocessed using FSL 6.0.7 (24) and MRtrix3 (25). Denoising using the MP-PCA method and Gibbs ringing removal were performed in MRtrix3. Eddy current–induced distortions and head motion were corrected using the FSL eddy tool. Because no reversed phase-encoding b0 images were acquired, TOPUP-based susceptibility-distortion correction was not performed. Skull and other nonbrain tissues were removed using bet2. Diffusion tensors were then fitted using the FSL dtifit tool to generate fractional anisotropy (FA), axial diffusivity (AD), radial diffusivity (RD), mean diffusivity (MD), and tensor component maps (Dxx, Dyy, and Dzz).

To ensure comparability across participants, the preprocessed FA images were aligned to the JHU-ICBM-FA standard template (26) using rigid-body and affine registration with FSL FLIRT, and the corresponding transformation matrices were applied to the tensor component maps. All preprocessing outputs underwent subject-level visual quality control for residual motion or eddy current artifacts, abnormal brain extraction, and registration failure. Images that did not meet the quality-control criteria were excluded.

#### 2.4.3 White matter microstructural assessment

After DTI preprocessing, peak width of skeletonized mean diffusivity (PSMD) was calculated according to a previously described method (7). The tract-based spatial statistics (TBSS) pipeline implemented in FSL was used. First, FA images from all participants were registered to the FMRIB58_FA standard template, from which a mean FA image was generated and the white matter skeleton was extracted. Individual MD maps were then projected onto the skeleton, and histogram analysis of the skeletonized MD values was performed. PSMD was defined as the difference between the 95th and 5th percentiles of the MD distribution within the white matter skeleton. PSMD values were expressed in units of 10^−4^ mm^2^/s. All FA registrations and white matter skeletons underwent visual quality control to confirm that the skeleton was centered on the major white matter tracts and to identify any apparent registration errors. Images that did not meet the quality-control criteria were excluded.

#### 2.4.4 Assessment of the DTI-ALPS index

The DTI-ALPS index was calculated using a previously reported automated pipeline (27). In this study, spherical regions of interest (ROIs; diameter, 5 mm) were automatically positioned bilaterally within the projection and association fiber regions at the level of the body of the lateral ventricles, adjacent to the medullary veins, based on color-coded FA maps. Diffusivities along the x-, y-, and z-axes were extracted from each ROI, and the DTI-ALPS index was calculated according to the formula proposed by Taoka et al. (11):

$$ALPSindex=\frac{mean(D_{xproj},D_{xassoc})}{mean(D_{yproj},D_{zassoc})}$$

Here, *D_xproj_* and *D_xassoc_* represent the diffusivities along the *x*-axis in the projection and association fiber regions, respectively; *D_yproj_* represents the diffusivity along the *y*-axis in the projection fiber region, and *D_zassoc_* represents the diffusivity along the z-axis in the association fiber region. ROI placement was fully automated, with no manual definition or adjustment. Two researchers, blinded to group allocation, independently inspected the automatically generated ROIs to assess anatomical correspondence and localization. Disagreements were resolved by consensus. Three participants (two with T2DM and one HC) were excluded because the automatically generated ROIs could not be reliably localized to the intended anatomical regions.

### 2.5 Statistical analysis

All statistical analyses were performed using IBM SPSS Statistics 27.0 (IBM Corp., Armonk, NY, USA) and R version 4.4.2. Normality of continuous variables was assessed using the Shapiro–Wilk test. Normally distributed continuous variables are presented as mean ± standard deviation and were compared using independent-samples t tests, whereas non-normally distributed variables are presented as median (interquartile range) and were compared using Mann–Whitney U tests. Categorical variables are presented as frequencies (percentages) and were compared using chi-square tests.

Normalized total CPV, total DTI-ALPS, and PSMD were defined as the primary imaging measures, whereas hemisphere-specific CPV and DTI-ALPS measures were considered secondary imaging measures. MMSE was analyzed as a cognitive screening measure. Group differences were assessed using general linear models (GLMs) adjusted for age, sex, BMI, and years of education. Model assumptions were assessed using Q-Q plots and Shapiro–Wilk tests of residuals, residual-versus-fitted plots, and Breusch-Pagan tests for heteroscedasticity. HC3 heteroscedasticity-robust standard errors were additionally used in sensitivity analyses.

Within the T2DM group, partial correlations were performed among diabetes duration, PSMD, normalized total CPV, total DTI-ALPS, and MMSE, controlling for age, sex, BMI, and years of education. This resulted in 10 pairwise associations. For these analyses, 95% confidence intervals (CIs) were estimated using 5,000 bias-corrected and accelerated (BCa) bootstrap resamples. The two associations with nominal *P* < 0.05 were further evaluated using leave-one-out sensitivity analyses.

For multiple-comparison correction, the eight group-comparison outcomes (normalized total CPV, left and right CPV, total DTI-ALPS, left and right DTI-ALPS, PSMD, and MMSE) were treated as one family and corrected using the Benjamini-Hochberg false discovery rate (FDR) procedure. The 10 within-T2DM partial correlations constituted a separate FDR family. The same eight-outcome family was used for the HC3 sensitivity analyses. Descriptive demographic and clinical comparisons were not included in the FDR correction.

No prospective sample-size calculation was performed because of the retrospective study design. A sensitivity power analysis indicated that, with 31 participants, four covariates, a two-sided *α* of 0.05, and 80% power, the minimum detectable partial correlation was approximately |r| = 0.50. All tests were two-sided. Statistical significance was defined as *P_FDR_* < 0.05 for FDR-corrected analyses and *P* < 0.05 otherwise.

## 3 Results

### 3.1 Demographic, clinical, and cognitive characteristics

A total of 62 participants were included, comprising 31 patients with T2DM and 31 healthy controls. The two groups were comparable in demographic characteristics, with no significant differences in age, sex, BMI, or years of education (all *P* > 0.05; Table 1). The median diabetes duration in the T2DM group was 10 years, and the median glycated hemoglobin (HbA1c) level was 8.80% (interquartile range, 7.70–10.40). Total intracranial volume (TIV) did not differ significantly between the two groups (*P* = 0.762).

### 3.2 Between-group differences in MRI measures and MMSE scores

General linear models (GLMs) were used to compare imaging measures and MMSE scores between the T2DM and healthy control groups, adjusting for age, sex, BMI, and years of education. Multiple comparisons were corrected using the Benjamini-Hochberg false discovery rate (FDR) procedure. For the primary imaging measures, patients with T2DM showed significantly greater normalized total CPV, lower total DTI-ALPS, and higher PSMD than healthy controls (all *P*_FDR_ < 0.05). Both left and right CPV were also significantly greater in the T2DM group in the secondary hemisphere-specific analyses (Table 2). MMSE scores were significantly lower in patients with T2DM (*P*_FDR_ < 0.001) (Table 2, Figure 3). Residual diagnostics indicated some departures from normality for the PSMD, left DTI-ALPS, and MMSE models, whereas no significant heteroscedasticity was detected in any model. Sensitivity analyses using HC3 robust standard errors yielded the same pattern of statistical significance as the conventional GLMs.

**Table 2:** Comparison of imaging measures and MMSE scores between the two groups.

Characteristic	HC (*n* = 31)	T2DM (*n* = 31)	*B (95% CI)*	*η*_Ρ_^2^	*P*	*P*_FDR_
Normalized total CPV	1.53 ± 0.46	2.00 ± 0.49	0.429 (0.188, 0.671)	0.185	<0.001	0.002
Normalized left CPV	0.82 ± 0.24	1.09 ± 0.29	0.251 (0.114, 0.388)	0.193	<0.001	0.002
Normalized right CPV	0.71 ± 0.24	0.91 ± 0.22	0.179 (0.061, 0.296)	0.142	0.004	0.007
Total DTI-ALPS	1.73 ± 0.16	1.57 ± 0.21	−0.112 (−0.205, −0.019)	0.095	0.019	0.021
Left DTI-ALPS	1.76 (1.62, 1.85)	1.56 (1.40, 1.74)	−0.098 (−0.203, 0.008)	0.058	0.069	0.069
Right DTI-ALPS	1.71 ± 0.16	1.54 ± 0.21	−0.126 (−0.221, −0.032)	0.113	0.010	0.013
PSMD	2.67 (2.40, 2.91)	2.99 (2.79, 3.20)	0.364 (0.109, 0.619)	0.127	0.006	0.010
MMSE score	28 (26, 29)	25 (23, 27)	−3.196 (−4.622, −1.769)	0.264	<0.001	<0.001

Continuous variables are presented as mean ± standard deviation or median (interquartile range). Between-group comparisons used general linear models adjusted for age, sex, BMI, and years of education. CPV measures were normalized as (CPV/TIV) × 10^3^ to account for individual differences in head size; the resulting normalized CPV values are dimensionless. PSMD values are expressed in units of 10^−4^ mm^2^/s. B, adjusted unstandardized regression coefficient; 95% CI, 95% confidence interval; *η*_Ρ_^2^, partial eta squared; *P*_FDR_, false discovery rate-adjusted *P* value; CPV, choroid plexus volume; TIV, total intracranial volume; DTI-ALPS, diffusion tensor image analysis along the perivascular space; PSMD, peak width of skeletonized mean diffusivity; MMSE, Mini-mental State Examination.

**Figure 3:**
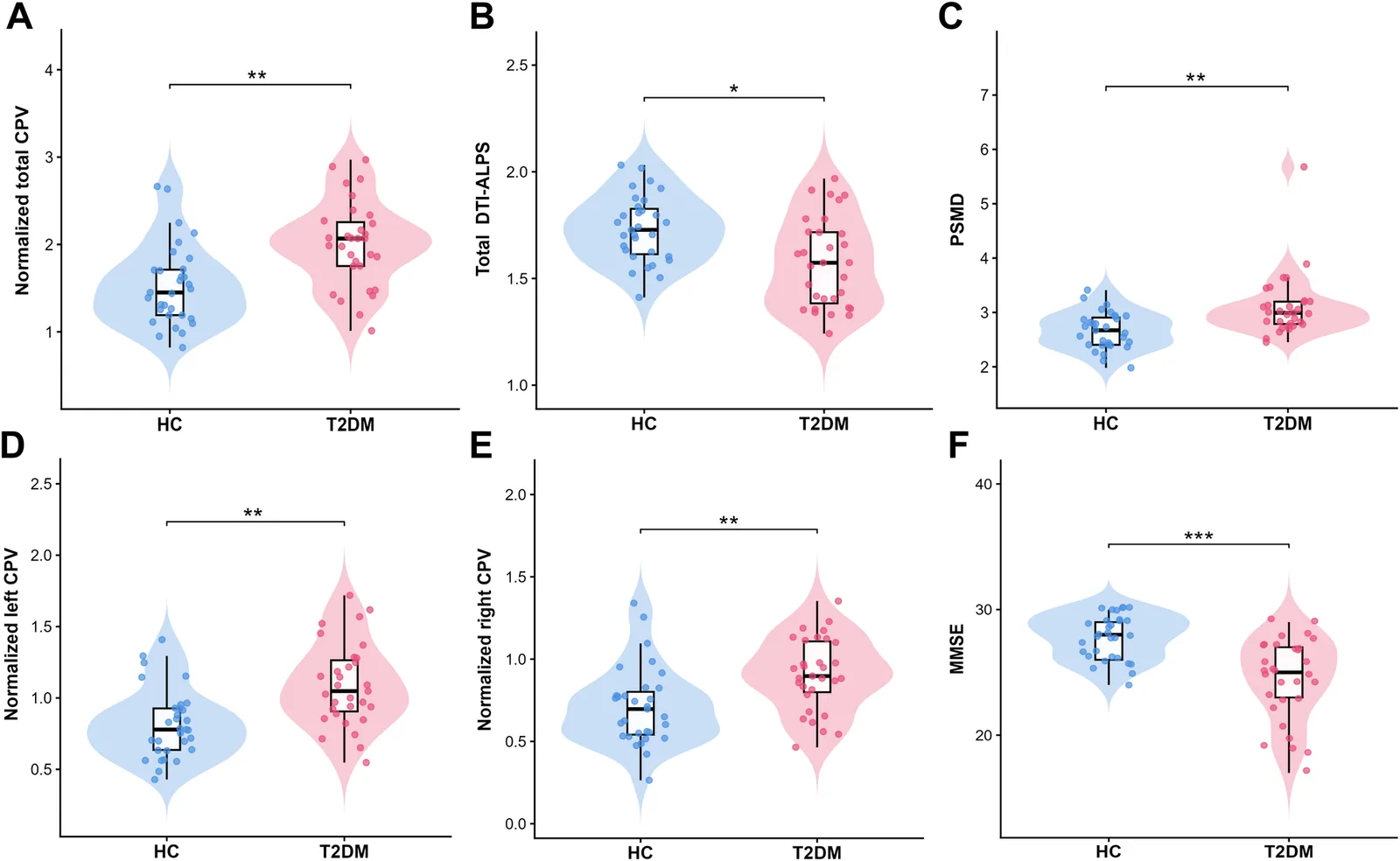
Violin plots of imaging measures and MMSE scores in the HC and T2DM groups. **(A)** Normalized total CPV; **(B)** Total DTI-ALPS; **(C)** PSMD; **(D)** Normalized left CPV; **(E)** Normalized right CPV; and **(F)** MMSE score. Points represent individual participants, and box plots indicate the median and interquartile range. Group comparisons were performed using general linear models adjusted for age, sex, BMI, and years of education. CPV measures were normalized as (CPV/TIV) × 10^3^. The resulting normalized CPV values are dimensionless. PSMD values are expressed in units of 10^−4^ mm^2^/s. All eight outcomes were corrected together using the Benjamini–Hochberg FDR procedure. *P_FDR_ < 0.05, **P_FDR_ < 0.01, and ***P_FDR_ < 0.001. CPV, choroid plexus volume; TIV, total intracranial volume; DTI-ALPS, diffusion tensor image analysis along the perivascular space; PSMD, peak width of skeletonized mean diffusivity; MMSE, Mini-Mental State Examination.

### 3.3 Exploratory association analyses in patients with T2DM

Within the T2DM group, exploratory partial correlation analyses were performed among diabetes duration, PSMD, normalized total CPV, total DTI-ALPS, and MMSE, adjusting for age, sex, BMI, and years of education. After FDR correction across the 10 pairwise associations, only the positive association between diabetes duration and PSMD remained significant (*partial r* = 0.566, *95% CI*, 0.154–0.793, *P* = 0.002, *P_FDR_* = 0.021). An inverse association between normalized total CPV and total DTI-ALPS was observed at the nominal level (*partial r* = −0.412, *95% CI*, −0.644 to −0.080, *P* = 0.033), but did not survive FDR correction (*P_FDR_* = 0.163). None of the remaining exploratory pairwise associations survived FDR correction (Table 3, Figure 4).

**Table 3:** Exploratory pairwise partial correlations in patients with T2DM.

Variable 1	Variable 2	*partial r (95% CI)*	*P*	*P*_FDR_
Diabetes duration	PSMD	0.566 (0.154, 0.793)	0.002	0.021
Diabetes duration	Normalized total CPV	−0.007 (−0.361, 0.382)	0.974	0.974
Diabetes duration	Total DTI-ALPS	−0.132 (−0.502, 0.238)	0.510	0.850
Diabetes duration	MMSE	0.021 (−0.352, 0.384)	0.916	0.974
PSMD	Normalized total CPV	0.107 (−0.237, 0.465)	0.595	0.850
PSMD	Total DTI-ALPS	−0.177 (−0.432, 0.150)	0.378	0.756
PSMD	MMSE	−0.026 (−0.380, 0.272)	0.898	0.974
Normalized total CPV	Total DTI-ALPS	−0.412 (−0.644, −0.080)	0.033	0.163
Normalized total CPV	MMSE	−0.267 (−0.577, 0.181)	0.179	0.596
Total DTI-ALPS	MMSE	0.188 (−0.274, 0.480)	0.348	0.756

Analyses were adjusted for age, sex, BMI, and years of education. Partial r values are presented with 95% CIs estimated using 5,000 BCa bootstrap resamples. The 10 pairwise associations were corrected together using the Benjamini–Hochberg FDR procedure. *P*_FDR_, FDR-adjusted *P* value; CPV, choroid plexus volume; DTI-ALPS, diffusion tensor image analysis along the perivascular space; PSMD, peak width of skeletonized mean diffusivity; MMSE, Mini-mental State Examination.

**Figure 4:**
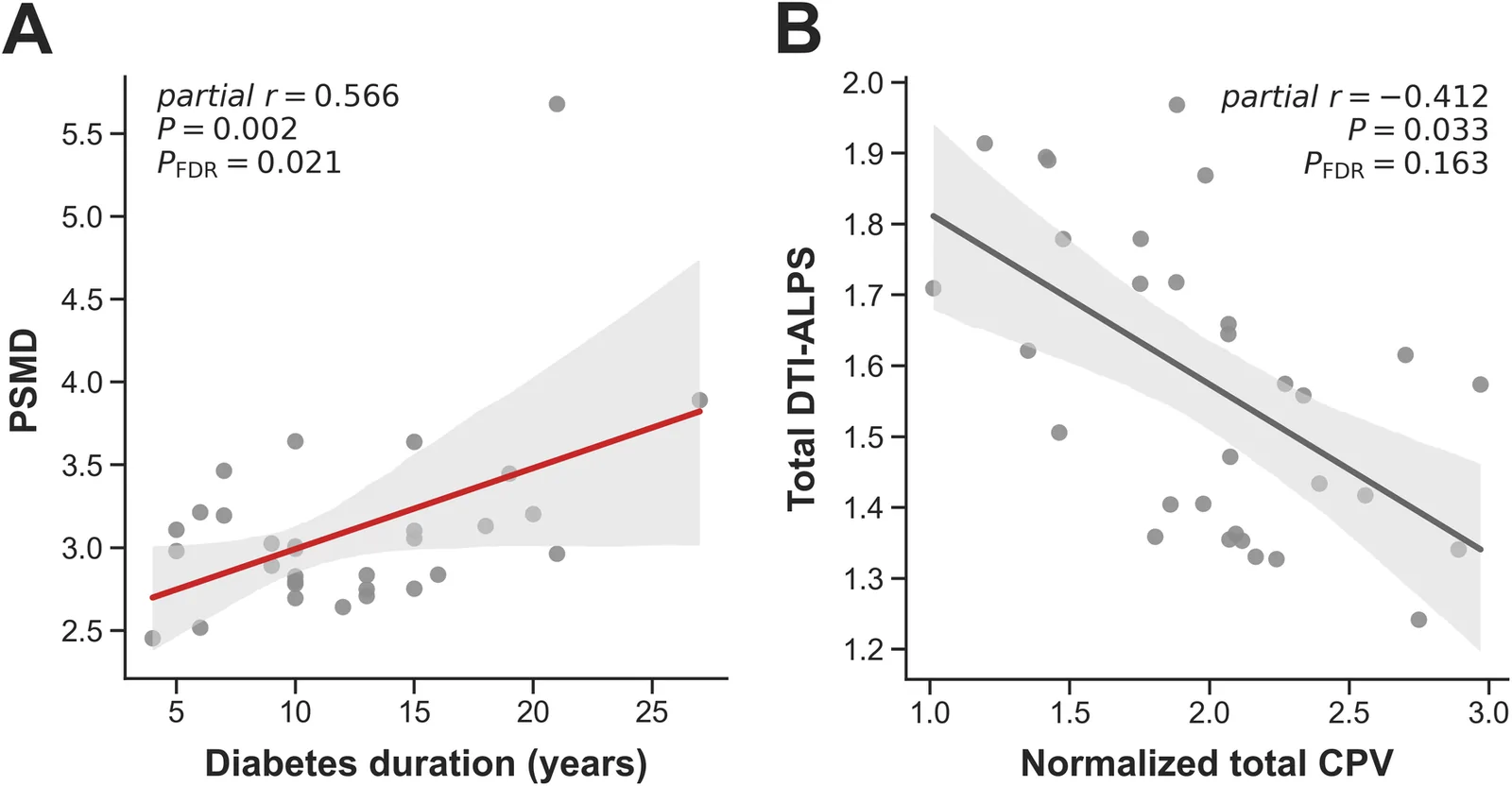
Exploratory partial correlations in patients with T2DM. **(A)** Positive association between diabetes duration and PSMD. **(B)** Inverse association between normalized total CPV and total DTI-ALPS. Partial correlations were adjusted for age, sex, BMI, and years of education. The 10 pairwise associations were corrected using the Benjamini–Hochberg FDR procedure. Solid lines indicate fitted trends and shaded areas represent 95% confidence intervals. *P*_FDR_, FDR-adjusted *P* value.

In leave-one-out sensitivity analyses, the direction of both associations remained unchanged, with partial r ranging from 0.436 to 0.611 for diabetes duration–PSMD and from −0.474 to −0.355 for normalized total CPV–total DTI-ALPS. After exclusion of the observation with the highest PSMD value, the diabetes duration–PSMD association remained positive and nominally significant (*partial r* = 0.436, *P* = 0.026).

## 4 Discussion

This study used multimodal MRI to characterize T2DM-related brain differences across choroid plexus structure, perivascular diffusion-related imaging characteristics, and white matter microstructure. Compared with healthy controls, patients with T2DM showed greater normalized total and bilateral CPV, lower total DTI-ALPS, higher PSMD, and lower MMSE scores. Within the T2DM group, longer diabetes duration was associated with higher PSMD after FDR correction, whereas the inverse association between normalized total CPV and total DTI-ALPS was nominally significant but did not survive FDR correction. Together, these findings indicate that T2DM-related brain differences involve multiple neuroimaging domains.

Higher PSMD in patients with T2DM is consistent with previous diffusion MRI evidence of widespread white matter microstructural abnormalities in diabetes. Prior studies have linked longer disease duration and poorer metabolic control to abnormalities across multiple DTI- and NODDI-derived measures (4–6). More recently, Tian et al. reported elevated PSMD in patients with T2DM and mild cognitive impairment and found a positive association between PSMD and diabetes duration (9), consistent with the present findings. Because PSMD reflects the dispersion of mean diffusivity values across the white matter skeleton, higher PSMD with longer diabetes duration may indicate greater whole-brain diffusivity heterogeneity. Chronic metabolic disturbance, cerebral microvascular dysfunction, and blood–brain barrier abnormalities may contribute to these white matter changes (2–4). Given the cross-sectional design, however, the association with disease duration should not be interpreted as evidence of longitudinal progression.

Greater CPV and lower DTI-ALPS were also observed in the T2DM group, in agreement with previous reports of increased CPV (18, 19) and reduced DTI-ALPS indices (9, 12, 15) in diabetes. The choroid plexus contributes to CSF production, maintenance of the blood–CSF barrier, and neuroimmune regulation (16, 17), whereas DTI-ALPS reflects directional diffusivity in perivascular white matter. These measures therefore capture distinct imaging features related to brain fluid homeostasis. The inverse association between normalized total CPV and total DTI-ALPS was nominally significant but did not survive FDR correction. Although a similar CPV–DTI-ALPS relationship has been reported in another disease context (20), the present finding should be considered preliminary. Shared vascular, barrier, or perivascular factors may contribute to this relationship (3, 28–30), but confirmation in independent cohorts is needed.

Patients with T2DM also had lower MMSE scores than healthy controls, consistent with previous reports of lower cognitive performance in T2DM (2, 3). However, the absence of known neurological, psychiatric, or neurocognitive comorbidities was based on available medical records and routine clinical history rather than dedicated diagnostic assessments. Therefore, unrecognized comorbidities cannot be excluded, and the cause of the lower MMSE scores cannot be determined with certainty. Notably, the very low MMSE scores occurred only in the T2DM group, but they could reflect T2DM-related factors, independent comorbidity, or both. In addition, the MMSE is a global cognitive screening tool and does not characterize domain-specific impairment or establish a clinical diagnosis (22, 31). Accordingly, the MMSE findings should be interpreted as a between-group difference in cognitive screening performance.

Taken together, the present findings support a multidimensional view of brain involvement in T2DM. White matter diffusivity heterogeneity, choroid plexus structure, and perivascular diffusion-related characteristics showed distinct group differences, while diabetes duration was specifically associated with PSMD after correction for multiple comparisons. Considering these measures together may provide a broader characterization of T2DM-related brain differences than any single imaging marker alone.

This study has several limitations. First, the cross-sectional design precludes causal or longitudinal inference. Second, the single-center design and small sample size limit generalizability and statistical power, particularly for the exploratory within-T2DM correlations. Third, detailed metabolic and vascular covariates and conventional cerebral small vessel disease markers could not be systematically assessed because of the retrospective design and the lack of suitable FLAIR imaging. Fourth, DTI-ALPS may be influenced by local fiber architecture, white matter abnormalities, vascular lesions, partial-volume effects, and residual susceptibility-induced EPI distortion. Reversed phase-encoding images were unavailable, precluding TOPUP-based correction. Finally, the exclusion of known neurological, psychiatric, and neurocognitive comorbidities relied on available medical records and routine clinical history rather than dedicated diagnostic assessments, and cognition was assessed only with the MMSE. Larger longitudinal studies incorporating more comprehensive clinical, neuropsychological, and imaging assessments are needed to validate and extend these findings.

## 5 Conclusion

This study identified T2DM-related differences in choroid plexus structure, perivascular diffusion-related imaging characteristics, white matter microstructure, and MMSE scores. Longer diabetes duration was associated with higher PSMD after FDR correction, whereas the inverse association between normalized total CPV and total DTI-ALPS did not survive multiple-comparison correction. These findings support a multidimensional characterization of brain involvement in T2DM and warrant validation in larger longitudinal cohorts.

## Data Availability

The data analyzed in this study is subject to the following licenses/restrictions: The datasets are not publicly available because of ethical and privacy restrictions on the sharing of participant-level clinical and MRI data. The data may be available from the corresponding author upon reasonable request, subject to approval by the relevant ethics committee and institutional requirements. Requests to access these datasets should be directed to Liyuan Fu, fu313870625@126.com.
